# Olfactory Signatures in the Food Finding Test in Mice With Normal and Alzheimer’s Disease-Pathological Aging With Special Concerns on the Effects of Social Isolation

**DOI:** 10.3389/fnins.2021.733984

**Published:** 2021-10-05

**Authors:** Daniela Marín-Pardo, Lydia Giménez-Llort

**Affiliations:** ^1^Institut de Neurociències, Universitat Autònoma de Barcelona, Barcelona, Spain; ^2^Department of Psychiatry and Forensic Medicine, School of Medicine, Universitat Autònoma de Barcelona, Barcelona, Spain

**Keywords:** neuroethology, 3xTg-AD mice, behavioral neuroscience, methods, smell loss, aging, Alzheimer’s disease, ethogram

## Abstract

The temporal course and the severity of the involution of sensory systems through aging can be critical since they ensure the ability to perceive and recognize the world. In older people, sensory impairments significantly increase their risk of biological, psychological, and social impoverishment. Besides this, olfactory loss is considered an early biomarker in Alzheimer’s disease (AD) neurodegenerative process. Here we studied olfactory ethograms in middle-aged male and female gold-standard C57BL/6 mice and 3xTg-AD mice, a genetic model of AD that presents cognitive dysfunction and a conspicuous neuropsychiatric-like phenotype. A paradigm involving 1-day food deprivation was used to investigate the ethological patterns shown in the olfactory inspection of a new cage and the sniffing, finding, and eating of hidden food pellets. The sniffing–find–eat temporal patterns were independent of the loss of weight and unveiled (fast) olfactory signatures in Alzheimer’s disease, differing from those (slow progressive) in normal aging. Male 3xTg-AD mice exhibited an early signature than female mice, opposite to animals with normal aging. The sequence of actions was correlated in male and female 3xTg-AD mice in contrast to control mice. Social isolation, naturally occurring in male 3xTg-AD due to the death of cage mates, emphasized their olfactory patterns and disrupted the behavioral correlates. The paradigm provided distinct contextual, sex, and genotype olfactory ethogram signatures useful to investigate olfactory function in normal and AD-pathological aging. Isolation had an impact on enhancing the changes in the olfactory signature here described, for the first time, in the 3xTg-AD model of Alzheimer’s disease.

## Introduction

Throughout life, sensory systems must ensure our ability to perceive and recognize the world. Thus, the time course and severity of its involution through the aging process are decisive for individuals since these limit the ability to sustain the quality of sensory stimuli, affecting not only cognitive processes but also their self-esteem, habits, and styles of a lifetime. In older people, sensory deficiencies significantly increase their risk of biological, psychological, and social impoverishment (Dziechciaz and Filip, 2014). Sensory deficits have been proposed as predictors of cognitive decline in older adults and as early indicators of the prodromal stages of Alzheimer’s disease. Among them, the association between olfactory dysfunction and cognitive impairment is of growing interest. Olfactory dysfunction has attracted much attention among researchers in the field of brain damage and is a well-known marker for many neurological diseases (Wilson et al., 2009). By causing such vulnerability and/or disability, sensory deficiencies significantly increase the risk of the impoverishment of older people on a biological, psychological, and social level (Volkers and Scherder, 2011). More importantly, emergent literature unveiled anosmia as being more predictive of 5-year mortality risk than cardiovascular disease (Van Regemorter et al., 2020).

The complete and adequate evaluation of patients plays a role in any diagnostic work to demonstrate the etiology, pathogenesis, diagnosis, and possible prevention/therapy in neurodegenerative disorders (Kondo et al., 2020). Olfactory loss, which is known to occur in bacterial and viral infections and is considered an early biomarker in the neurodegenerative processes of Alzheimer’s and Parkinson’s diseases (Kovács, 2004), has also been reported as an early indicator of current SARS-CoV-2 infection (Mahalaxmi et al., 2020). Sniffing impairment or deficiency is present in almost 90% of the population with Alzheimer’s disease (AD) (Conti et al., 2013). Furthermore, in certain cases with mild cognitive impairment, this loss has also been observed during the transition phase between normal aging and dementia. Previous studies have found that olfactory impairment or deficiency may be an indicator that people affected by mild cognitive impairment are more likely to develop dementia (Invitto et al., 2018). A first diagnostic screening based on olfactory tests would allow the rapid implementation of preventive approaches and thus improve cognition and brain and mental health (Brai et al., 2020).

Male and female sexes can be regarded as two exceptional natural scenarios to study the role of biological, psychological, and social factors, their functional interplay, and their impact on the crosstalk of homeostatic networks in health and disease through life cycle (Giménez-Llort et al., 2014). At the translational level, in the present brief report, we have studied the olfactory signatures in male and female mice with normal and neurodegenerative aging associated with advanced stages of AD. For this purpose, we used 3xTg-AD mice (Oddo et al., 2003) and age-matched non-transgenic mice, both with a C57BL/6J genetic background (Baeta-Corral and Giménez-Llort, 2014). The 3xTg-AD mouse is a genetic model of Alzheimer’s disease that presents not only AD cognitive dysfunction but also a striking phenotype modeling some “behavioral and psychological symptoms of dementia” (BPSD), including neuropsychiatric symptoms such as anxiety, apathy, and depression-like behaviors (Giménez-Llort et al., 2006, 2007; Torres-Lista and Giménez-Llort, 2013; Baeta-Corral et al., 2018a).

In accordance with the increased mortality rates in dementia compared to the older population (van Dijk et al., 1991), the mortality of 3xTg-AD mice is higher than that of their wild-type counterparts of the same age, a phenomenon that has been related to their neuro-immune-endocrine impairment (Giménez-Llort et al., 2008). Furthermore, in male mice, the rates increase exponentially at 12 to 13 months (Giménez-Llort et al., 2008), an age mimicking the advanced stages of the disease (Oddo et al., 2003; Belfiore et al., 2019), thus providing a naturalistic scenario to study the impact of the loss of life mates.

## Materials and Methods

### Animals

A total number of 97 13-month-old male and female homozygous 3xTg-AD (*n* = 58, 22 male and 36 female) and non-transgenic (NTg, *n* = 39, 24 male and 15 female) mice on a C57BL/6J background (after embryonic transfer and backcrossing at least 10 generations) established in the Universitat Autònoma de Barcelona (Baeta-Corral and Giménez-Llort, 2014) were used in this study. The 3xTg-AD mice harboring transgenes were genetically engineered at the University of California Irvine, as previously described (Oddo et al., 2003). The animals were maintained under standard laboratory conditions of food and water *ad lib*, 22 ± 2°C, 12-h light/dark cycle with lights on at 8:00 am, and relative humidity of 50–60%.

#### Social Conditions

Animals of the same genotype and sex were maintained in groups of three to four mice per cage (Macrolon, 35 cm × 35 cm × 25 cm), which was filled with 5 cm of clean wood cuttings (Ecopure, Chips6, DateSand, United Kingdom; Uniform cross-cut wood granules with 2.8–1.0 mm chip size) and nesting materials (Kleenex, Art: 08834060, 21 cm × 20 cm, White). In the current work, a *natural social isolation scenario* was found among male 3xTg-AD mice due to increased mortality rates in male mice of this genotype occurring at this age (Giménez-Llort et al., 2008). Thus, short isolation (2–3 months) was observed in seven of the 22 male 3xTg-AD that recently lost their cage mates after 10 months of living in a standard social environment. In all the cases, the standard home cages covered with a metallic grid allow the perception of olfactory and auditory stimuli from the rest of the colony.

### Behavioral Assessment

#### Physical Status

To monitor the effects of fasting used in the food finding paradigm, body weight was measured before (Wpre) and after (Wpost) food deprivation. The recovery of weight was monitored at 24 h later (Wfinal).

#### The Food Finding Test of Olfactory Ability

To investigate olfactory function, the food finding test (FFT) olfactory paradigm (Deacon et al., 2009) was used. The mice were deprived of food for 14 h before testing (starting with lights off at 2000h), the equivalent to 1-day food deprivation.

The test was performed under dim white light (20 lx) during their light phase of the light/dark cycle (from 10:00 to 11:00 a.m.) using a novel cage (50 cm × 22 cm × 14 cm) with 1 cm of beddings. Eight food pellets (45 mg) were placed in the central zone of the cage and covered by 1 cm of wood chip bedding. The mouse was placed in a corner of the cage, facing the walls, and its behavior was observed. The latencies of three goal-directed behaviors toward hidden food were recorded, namely, sniffing—when the nose makes a direct contact with a surface and the vibrissae are in maximum extension and are in contact with the surface in the same way (Welker, 1964); finding (digging)—“finding a food pellet” was defined as digging, touching, and holding the pellet in the front paws for more than 3 s; and eating the hidden food—holding the pellet in the front paws for more than 3 s of continuous eating. The beddings were renewed between each mouse.

Behavioral assessments were performed in a counterbalanced manner by direct observation by an observer blind to the genotype and the support of video recordings (ViewPoint Behavior Technology, Lion, France) for complementary retrospective analysis. All procedures followed the Spanish legislation on the “Protection of Animals Used for Experimental and Other Scientific Purposes” and the EU Directive (2010/63/UE) on this subject. The study complies with the ARRIVE guidelines developed by the NC3Rs and aims to reduce the number of animals used (Kilkenny et al., 2010).

### Statistical Analysis

The results are expressed as mean ± SEM. SPSS 20.0 software was used. A 2 × 2 factorial design was used to analyze the effects of (G) genotype and (S) sex factors. Differences were studied through multivariate general linear model analysis, followed by *post hoc* Duncan’s test (multiple comparisons). In analyzing the effects of social isolation, the differences between two independent groups were measured with Student’s *t*-test. In all the tests, *p* < 0.05 was considered statistically significant.

## Results

### Effect of Food Deprivation on Body Weight but Not Related to the Olfactory Signature

**FIGURE 1:**
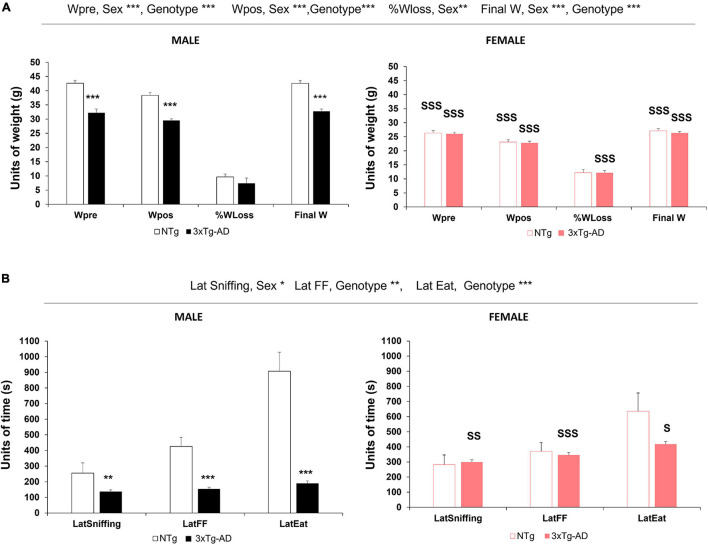
Genotype and sex differences in body weight monitoring and food finding test in 13-month-old NTg and 3xTg-AD mice. The results are expressed as mean ± SEM. **(A)** Food deprivation: Wpre, initial weight before fasting; Wpost, weight after fasting;%Wloss, percent of weight lost overnight; Final W, final weight at 24 h later. **(B)** Food finding test—latencies in the food finding test: LatSniffing, latency to sniffing the hidden pellet; LatFF, latency to find the food (hidden pellet); LatEat, latency to eat the pellet. Statistics: **p < 0.01, ***p < 0.001 *vs.* NTg mice of the same sex; S, *p* < 0.05; SS, *p* < 0.01; SSS, *p* < 0.001 *vs*. male of the same genotype.

The body weight of animals was monitored throughout experimental procedures (Figure 1A). At the beginning of the experiments, before the animals were submitted to food deprivation for 14 h, the weight of 3xTg-AD mice was lower than that of controls [Wpre, genotype^∗∗∗^, *F*(1, 86) = 34.369, *p* < 0.001]. After the food finding test, all the groups of animals lost weight, but genotype differences persisted [Wpos, genotype^∗∗∗^, *F*(1, 86) = 36.481, *p* < 0.001]. Sexual dimorphism was found in the initial body weight [Wpre, sex^∗∗∗^, *F*(1, 86) = 152.694, *p* < 0.001]. Thus, while the weight of male 3xTg-AD mice was lower than C57BL/6J, the body weight of female mice was independent of the genotype. As expected, food deprivation did not modify sexual dimorphism [Wpos, sex^∗∗∗^, *F*(1, 86) = 210.954, *p* < 0.001]. Regarding the percentage of weight loss [%Wloss, sex^∗^, *F*(1, 86) = 6.585, *p* < 0.05], it was in the range of 5–10% of their weight, independently of the genotype but higher in female mice than in male mice in the 3xTg-AD genotype. At 24 h after fasting, the animals of both genotypes and sexes almost regained their initial weight and showed genotype [Final W, genotype^∗∗∗^, *F*(1, 86) = 52.252, *p* < 0.001] and sex [Final W, sex^∗∗∗^, *F*(1, 86) = 223.375, *p* < 0.001] differences.

The effects of food deprivation and the food finding test in the male sex indicated that the loss of weight, expressed either in units of weight or as a percentage, was not related to the statistically significantly shorter latency of 3xTg-AD mice to find the food and to eat it.

### Sex- and Genotype-Dependent Signatures in the Food Finding Test

As indicated in Figure 1B, the FFT paradigm to investigate the olfactory function in normal and AD-pathological aging elicited different sex- and genotype-dependent olfactory ethogram signatures. There was a significant genotype difference between the 3xTg-AD and NTg animals regarding the three actions, albeit only finding [Lat FF, genotype^∗∗∗^, *F*(1, 86) = 12.686, *p* < 0.001] and eating the food [Lat Eating, genotype^∗∗∗^, *F*(1, 86) = 41.984, *p* < 0.001] reached statistical significance. Sex differences were found between male mice (faster) and female mice (slower) just in the actions of sniffing [Lat Sniffing, sex^∗^, *F*(1, 86) = 5.558, *p* < 0.05], but not in the food finding and eating action [Lat FF, n.s., *F*(1, 86) = 2.811, *p* = 0.097; Lat Eating, n.s., *F*(1, 86) = 0.131, *p* = 0.718].

Male 3xTg-AD mice were faster in all the actions compared to NTg mice, with statistically significant differences in the latency of finding the hidden food [male, Lat FF^∗∗∗^, *F*(3, 86) = 8.716, *p* < 0.001] and eating it once it was found [male, Lat Eat^∗∗∗^, *F*(3, 86) = 17.064, *p* < 0.001], while those in the latency to sniffing the food did not reach statistical significance.

Moreover, very short delays between actions in the male 3xTg-AD mice indicated an immediate execution of actions, which differed from the progressive time increase from one action to the other exhibited by male NTg mice. Besides this, the delays between actions also indicated a 10-fold faster time pattern in male 3xTg-AD mice than NTg mice (Table 1).

**TABLE 1:** AD-genotype and sex effects in the time delay between actions.

	**NTg, male**	**NTg, female**		**3xTg-AD, male**		**3xTg-AD, female**	
**Time delay (s)**	**(*n* = 24)**	**(*n* = 15)**		**(*n* = 15)**		**(*n* = 36)**	
	**Mean ± SEM**	**Mean ± SEM**	**Ratio S**	**Mean ± SEM**	**Ratio G**	**Mean ± SEM**	**Ratio S**
Sniffing—finding	173.27 ± 45.5	87.93 ± 27.1	1/2 ×	16.75 ± 4.9***	10×	47.06 ± 19.5	2×
Finding–eating	488.13 ± 134.8	265.13 ± 28.4	1/2×	40.58 ± 13.8***	10×	71.67 ± 43.2	3×
Sniffing—eating	661.40 ± 137.6	353.07 ± 38.4^S^	1/2×	57.33 ± 13.7***	10×	118.72 ± 47.9*	2×

*The results are expressed as mean ± SEM. Time delay (s) between actions: sniffing and finding food, finding food and eating it, and sniffing and eating it.*

*Ratio S, fold increase vs. male mice of the same genotype; Ratio G, fold increase vs. male NTg mice.*

*p < 0.05, ***p < 0.001 vs. NTg mice of the same sex; S, p < 0.05 *vs*. mice of the same genotype.

**FIGURE 2:**
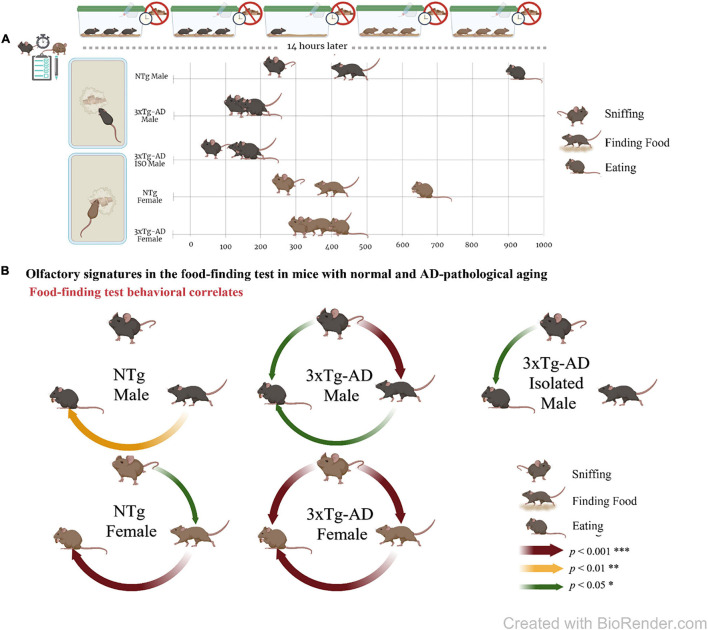
Olfactory signatures in mice with normal and AD-pathological aging in the food finding test. **(A)** Experimental design and ethogram tablature for “sniffing, finding, and eating the hidden food pellet” by 13-month-old male and female 3xTg-AD and NTg mice and natural isolation male 3xTg-AD mice. **(B)** Meaningful correlation analysis in food finding test. Graphical representation of the significant Pearson *r* correlations between the three olfactory actions. All of them were positive. The figure was created with BioRender.com.

In female mice, both groups exhibited longer latencies compared to male mice, and the time pattern of their ethogram for sniffing–finding–eating food was slower [Figure 2, female, LatSniffing, S, *p* < 0.05; LatFF, SSS, *p* < 0.001; LatEat, SS, *p* < 0.01]. However, the time delay between actions in the female 3xTg-AD mice was very short, as described for male 3xTg-AD mice, but with two- to threefold increase compared to them. In female NTg, the delays were shorter than those of male NTg, with values that were half of those recorded for them (see Table 1). Therefore, the male–female ratios in NTg and 3xTg-AD mice were in an opposite sense.

### Food Deprivation and Food Finding Test in Naturalistic Social Isolation of Male 3xTg-AD Mice

As detailed in Table 2, the naturalistic social isolation of 3xTg-AD mice did not result in changes in the temporal patterns compared to group-housed mice of the same genotype. On the other hand, the genotype-dependent statistical differences in the ethogram were enhanced in the isolated group, thus the latency of finding the hidden food [Table 2, Lat FF, genotype^∗∗∗^, *F*(2, 43) = 20.275, *p* < 0.001] and eating the food [LatEating, genotype^∗∗∗^, *F*(2, 43) = 10.930, *p* < 0.001].

**TABLE 2:** Effects of naturalistic isolation of male 3xTg-AD mice in food deprivation, food finding test, and the time delay between actions.

	**NTg, male**		**3xTg-AD, male**	**Ratio G**	**3xTg AD-ISO, male**	**Ratio G**
	**(*n* = 24)**		**(*n* = 15)**		**(*n* = 7)**	
	**Mean ± SEM**		**Mean ± SEM**		**Mean ± SEM**	
**Food deprivation**						
Wpre (g)	42.63 ± 1.00		32.23 ± 1.06***		30.2 ± 0.82***	
Wpos	28.49 ± 1.11		29.53 ± 0.48		28.19 ± 0.75	
%Wloss (g)	6.92 ± 2.50		7.40 ± 1.51		6.66 ± 0.28	
Final W (g)	31.74 ± 0.91		32.77 ± 0.65***		31.33 ± 0.75***	
**Food finding test**						
Latency of sniffing (s)	251.00 ± 63.88		135.71 ± 16.28		85.14 ± 14.58	
Latency of finding food (s)	424.27 ± 57.92		152.46 ± 15.79		111.86 ± 17.16***	
Latency of eating (s)	912.40121.02		193.04 ± 17.80		138.14 ± 23.62***	
**Time (s) delay**						
Sniffing—finding	173.27	45.50	16.75 ± 4.90***	10×	26.71 ± 10.77**	6×
Finding—eating	488.13	134.81	40.58 ± 13.85***	10×	26.29 ± 14.97***	19×
Sniffing—eating	661.40	137.57	57.33 ± 13.75***	10×	53.00 ± 12.92***	12×

*The results are expressed as mean ± SEM. Time (s) between actions: sniffing and finding food, finding food and eating it, and sniffing and eating food.*

*Wpre, initial weight before fasting; Wpost, weight after fasting;%Wloss, percent of weight lost overnight; Final W, final weight at 24 h later; LatSniffing, latency to smell the hidden pellet; LatFF, latency to find the hidden pellet; LatEat, latency to eat the pellet; Ratio G, fold increase *vs*. male NTg mice.*

**p < 0.01, ***p < 0.001.

### Correlation Analysis Between the Actions of Olfactory Signatures in the Food Finding Test

As shown in Figure 2B, the meaningful correlation analysis evidenced that, in the 3xTg-AD mice, the actions were closely related and predictable, mostly in female mice where statistical correlation increased in magnitude. In male mice living in naturalistic isolation conditions, the latency of sniffing was predictive of eating, but not of finding the food, and some of these correlations were lost. In male NTg mice, correlations were found between the finding and eating for male mice, and correlations were enhanced in order of magnitude in female mice.

## Discussion

The present work characterizes AD genotype and sex effects on olfactory ethograms in the food finding test that define olfactory signatures in male and female non-transgenic and 3xTg-AD mice.

The food finding test (Deacon et al., 2009) is an easy and ready-to-do test that can be used in any animal department setting without the need to buy anything since it is based on the ethological response of animals to fasting. Therefore, using this behavioral paradigm also allows doing a neuroethological assessment of sniffing function. Approaches that really take into account the specific characteristics of the behavior of the species and use ethological methods will be the most useful to interpret these behavioral findings and to better understand the biological mechanisms in brain function. The use of ethologically relevant stimuli is of particular importance to assess cognitive performance and dissect the effects of preventive/therapeutical interventions or the impact of risk factors and hazards (Torres-Lista et al., 2015, 2019; Giménez-Llort and Torres-Lista, 2021).

Throughout the experimental process in the food finding test, weight is monitored to assess the impact of food finding (Deacon et al., 2009). In the context of aging, body weight also serves as a good indicator of the health status of the animal (Barron et al., 2013). In the case of 3xTg-AD mice, we have shown that weight loss depends on intrinsic factors such as increased nocturnal activity (Baeta-Corral et al., 2018b) and hypermetabolic state (Escrig et al., 2020) compared to age-matched NTg mice. We have also shown that weight loss is useful to monitor the aggravation of the AD-pathogenic processes (Torres-Lista et al., 2019).

The exploratory behavior implies an internal conflict between the attraction for the novelty of space and the risk it contains since they could be exposing themselves to potentially dangerous environments (Giménez-Llort et al., 1995). In the case of rodents, most of this behavior depends on olfaction, a sense that allows not only the detection of odors in the surrounding environment necessary to identify food, predators, and sexual partners but also to get the spatial orientation (Invitto et al., 2018; Coronas-Sámano et al., 2014) being indispensable for survival. Once the animals had inspected the arena, the latencies to sniffing, finding, and eating the food pellets were found to have progressively increased in male mice with normal aging but consecutively developed in 3xTg-AD mice. It is known that olfactory deterioration during normal aging implies the loss of different olfaction skills, such as recognizing hidden food odor (Murphy et al., 1997; Schiffman, 1997). As referred to in the “Introduction”, recent literature on olfaction as a reflection of general health status also unveil a strong link between olfactory impairment and mortality, with underlying mechanisms being suspected to be associated with the impact of olfactory loss on intrinsic (nutrition, accelerated brain aging, and neurodegenerative diseases) and extrinsic (life-threatening situations and social interactions) mechanisms (Van Regemorter et al., 2020).

In the present work, male mice with normal and AD-pathological aging were equally delayed in their first contact with food pellets, while in the female sex this latency was increased but also depended on the genotype. In contrast, female 3xTg-AD performed the three-action (sniffing–find–eat) ethogram compared to their males cage mates. This could be because the sniffing in female mice is prone to be more affected during this stage of the disease, developing a situation in which the sensitivity to detect odors is more deficient (Mitrano et al., 2021).

Sex differences were found in the latency of the actions, with female mice being delayed compared to male mice, and also in the delay to develop the sequence, in this latter case being in a genotype-dependent manner. Thus, both groups of female mice exhibited longer delays in starting the ethogram than male mice, but the temporal pattern of their ethogram (delays between actions) to sniffing–finding–eating the food was two- to threefold times faster in the 3xTg-AD mice, but 1/2 in the case of NTg mice. This paradoxical genotype and sex modulation of the ethogram could be described as an accordion.

In the present work, the effects of naturalistic isolation (2–3 months) in a subgroup of 3xTg-AD that recently lost their cage mates after 10 months of living in a standard social environment were also assessed. This short isolation was enough to enhance the AD-genotype olfactory patterns shown by group-housed mice of the same genotype. When bearing different explanatory hypotheses, it is important to note that the body weight and the loss of weight after overnight fasting of isolated male 3xTg-AD were similar to those in the group-housed male 3xTg-AD, suggesting similar food motivation. The current report also shows that the ethogram was independent of body weight. Another explanation for their ability to find the hidden food faster could be related to a more intact olfactory function to detect food and recognize it over the other cage smells. In this case, the results would also suggest these mice as being somehow resilient to insults upon their nervous system, and according to the strong link between olfaction loss and mortality (Van Regemorter et al., 2020), this could explain why they lived longer than their cage mates. However, 100% of the group-housed 3xTg-AD mice could also be considered survivors with respect to the mean life span of the colony, and the female 3xTg-AD mice—that have better survival than male mice—were delayed compared to male mice. The most plausible interpretation could be that the shorter “inspection” of the new environment performed by 3xTg-AD mice living in social conditions is related to their anxiety-like profile and reduced to a minimum inspection in the isolated animals. In fact, we have recently reported increased anxiety in naturalistic isolated 3xTg-AD mice (Muntsant and Giménez-Llort, 2020) and distinct digging signatures, an ethological behavior of animals whose enhancement in the 3xTg-AD models neuropsychiatric-like behaviors associated with AD (Giménez-Llort and Alveal-Mellado, 2020) and where the olfactory function is also involved. However, since female 3xTg-AD are more anxious than male 3xTg-AD, other neuronal mechanisms may also be underlying these behavioral observations.

The time interval between actions to inspect the beddings was 10 times faster in the 3xTg-AD animals than in the NTg mice with normal aging. The control animals confirm that the sense of sniffing deteriorates during normal aging, and the different signatures that characterize sniffing, such as the detection of hidden pellet odor, are lost.

Meaningful correlations between the three actions in the food finding test indicate that the actions were correlated in the AD-genotype as could be inferred because the temporal pattern consisted of actions immediately consecutively performed. In the case of 3xTg-AD mice, the accordion effect resulted in correlations with a stronger statistical significance. Interestingly, the lower number of correlations found in the NTg mice, despite the bigger sample size in this group, would suggest that behavioral patterns driven by olfactory function coexisted with behaviors driven by other cognitive functions. It is important to note that the variables were independent of the loss of body weight. In isolated 3xTg-AD mice, the correlations were mainly lost, but we cannot discard this due to the small sample size limitation.

In summary, the present results on the olfactory signatures in male and female 3xTg-AD mice compared to C57BL/6 non-transgenic mice show the following: (1) the sniffing–find–eat temporal pattern was independent of the loss of weight, (2) olfactory signatures in male and female 3xTg-AD mice were characterized by a fast ethogram and strong behavioral correlates, (3) olfactory signatures in male and female controls, the gold-standard C57BL/6 mice, were slow and progressively developed as part of a more complex ethogram, as also supported by disrupted behavioral correlates between the three actions assessed in the food finding test, (4) social isolation, naturally occurring only in 3xTg-AD mice, enhanced the genotype differences and disrupted the behavioral correlates.

## Data Availability Statement

The raw data supporting the conclusions of this article will be made available by the authors, without undue reservation.

## Ethics Statement

The animal study was reviewed and approved by CEEAH/Universitat Autònoma de Barcelona and DARP/Generalitat de Catalunya.

## Author Contributions

LG-L: conceptualization. DM-P: performance and analysis of behavior, illustrations and draft manuscript. Both authors equally contributed to the scientific discussions, the edit writing, and approval of the manuscript.

## Conflict of Interest

The authors declare that the research was conducted in the absence of any commercial or financial relationships that could be construed as a potential conflict of interest.

## Publisher’s Note

All claims expressed in this article are solely those of the authors and do not necessarily represent those of their affiliated organizations, or those of the publisher, the editors and the reviewers. Any product that may be evaluated in this article, or claim that may be made by its manufacturer, is not guaranteed or endorsed by the publisher.
