# Supplementary material for: Olfactory Signatures in the Food Finding Test in Mice With Normal and Alzheimer’s Disease-Pathological Aging With Special Concerns on the Effects of Social Isolation
Source: Front Neurosci. 2021 Oct 5;15:733984. doi: 10.3389/fnins.2021.733984 (PMC8523944; doi:10.3389/fnins.2021.733984)
Supplement: Supplementary file 1 [file Table_1.pdf]

frontiers

Olfactory signatures in the food-finding test in male and female mice with normal and AD-pathological aging with special concerns on the effects of social isolation

Daniela Marín-Pardo, Lydia Giménez-Llort

Supplementary Table 1. Factorial analysis

Variables	F	Factor Sex	Factor Genotype	Significance p value
**A. Food deprivation**				
Wpre	F(1,86)= 34.369		G***	p= 0.000
	F(1,86)= 152.694	S***		p= 0.000
Wpos	F(1,86)=36.481		G***	p= 0.000
	F(1,86)=210.954	S***		p= 0.000
%Wloss	F(1,86=6.585	S*		p= 0.012
Final Weight	F(1,86)=52.252		G***	p= 0.000
	F(1,86)=223.375	S***		p= 0.000
**B. Food-finding test**				
Latency of Smell	F(1,86)=5.558	S*		p= 0.021
Latency of Finding Food	F(1,86)=12.686		G***	p= 0.001
Latency of Eating	F(1,86)=41.984		G***	p= 0.000
**Time delay between actions**				
Smelling- Finding	F(1,86)=15.535		G***	p= 0.000
Finding – Eating	F(1,86)=28.649		G***	p= 0.000
Smelling – Eating	F(1,86)=44.539		G***	p= 0.000
	F(1,86)=3.864	(S*)		p= 0.053
**Naturalistic Isolation**				
**A. Food deprivation**				
Wpre	F(2,43)= 30.546		G***	p= 0.000
Wpos	F(2,43)=44.698		G***	p= 0.000
Final Weight	F(2,43)=52.114		G***	p= 0.000
**Food-finding test**				
Latency of Smell	F(2,43)=3.661		G*	p= 0.034
Latency of Finding	F(2,43)=20.275		G***	p= 0.000
Latency of Eating	F(2,43)=34.979		G***	p= 0.000
**Time delay between actions**				
Smelling- Finding	F(2,43)=11.517		G***	p= 0.000
Finding – Eating	F(2,43)=10.930		G***	p= 0.000
Smelling – Eating	F(2,43)=19.130		G***	p= 0.000

Abbreviations: Wpre, initial weight before fasting; Wpost, weight after fasting; % Wloss, % weight lost overnight; Final W, final weight 24h later. Statistics: G, genotype, * p < 0.05, ** p < 0.01, ******* p < 0.001 *vs.* NTg mice of the same sex; S, sex, * p < 0.05, ** p < 0.01, *** p < 0.001 vs. male of the same genotype.
